# Stable Bipolar Patient Switched to Mania following Clinical Doses of Prednisone

**DOI:** 10.1155/2011/797658

**Published:** 2011-10-19

**Authors:** Vikram Panwar, Krishan Lassi

**Affiliations:** Department of Psychiatry, Pawnee Mental Health Services, 2001 Claflin Road, Manhattan, KS 66502, USA

## Abstract

We describe a stable bipolar female patient on medications that got switched into mania due to higher doses of prednisone prescribed for her severe sinusitis.

## 1. Introduction

Steroids have been prescribed for a long time for variety of clinical conditions (Immunological diseases, cancers, respiratory conditions, and musculoskeletal conditions). Approximately, ten million steroid prescriptions are issued every year in USA [1]. Studies have reported three types of psychiatric adverse effects in patient on steroid therapy: (1) mild, nonpathological euphoria, (2) clinically significant psychiatric symptoms, (3) bipolar disorder induced by course of steroids [2, 3]. Various reports have shown that incidence of steroid psychosis is between 5%–20%. Most patients develop symptoms within days to weeks of starting treatment [1, 4]; but symptoms can occur any time after stopping the steroids [5]. We present a case of 44-year-old female with a history of stable bipolar disorder who developed manic symptoms when started on prednisone for her severe sinusitis. She was stable on medicines for bipolar disorder for quite some time.

## 2. Case

A 44-year-old, unemployed, married woman, living with her husband and 2 daughters was presented to the clinic with a history of mood swings and psychosis. She reported of having depressed mood, decreased energy, and a feeling of guilt and hopelessness for the last one week. She denied having any suicidal or homicidal ideations. The patient stated that she was diagnosed with bipolar II disorder at the age of 22 years; her symptoms were always mild and did not interfere with her activities. During her hypomanic phase she endorsed decrease in sleep, increased involvement in activities, high energy level, and impulsive activities (shopping spree). According to her in last few years her symptoms have increased in intensity and progressed into mania (delusions, illusions, auditory hallucinations, and obsessions). During manic period she did a lot of impulsive acts. During her most recent episode, she got obsessed with a person whom she felt she was in love with. She sent him text messages and followed him home; this continued for weeks. She was also involved in high-speed chase with cops and got into lot of trouble with law and family and had to be hospitalized. Three weeks prior to her hospitalization she was treated for her sinusitis with 60 mg oral prednisone. She reported of having a similar switch from hypomania to mania in past on using prednisone for her sinusitis. In the hospital, her steroids were tapered 5 mg every 4 days, and her symptoms were resolved.

She did not report any drug allergy or any kind of abuse during her childhood. Her family history was not known as she was adopted and did not know her biological parents. She used to work full time but was currently unemployed and was also in the process of divorce. She denied smoking, abusing, alcohol, or use of any recreational drugs. Her current medications are Trazodone (150 mg QHS), Risperidone (1 mg QAM), Zyprexa (15 mg QHS), and Lithium (300 mg TID).

On examination, the patient appeared her stated age and was clean with appropriate clothing. Her posture was appropriate and had normal body movement and speech. Her behavior was cooperative and had intact cognitive functions. She denied any perceptual disturbances currently, and her thought process was normal. She had a good insight about her mental illness but poor judgment as she had poor personal relationships.

Laboratory workup failed to reveal any significant abnormalities.

## 3. Discussion

Steroids have been widely prescribed for a long time for a variety of clinical conditions. Since 1950s, side effects of steroids have been well documented. Early side effects include insomnia, excitation, and distractibility followed by depression, mania, hypomania, and in some cases psychosis [2]. Studies have reported that majority of patients develop these symptoms within 3-4 days; but symptoms can occur as late as 3 weeks [6]. Most of the patients recover within 5-6 weeks after tapering of steroids but it may take longer in some cases [1, 6]. The cause of steroids side effects is not clearly understood, but it is believed to be due to cholinergic and dopaminergic stimulation [7]. Research has shown that steroids can alter sodium potassium pump and ion flux across membrane effecting norepinephrine metabolism particularly in reticular activating system [7, 8]. Various case reports show that there is linear dose response relationship; higher the dose (>40 mg/day) the more the chances of developing psychotic episodes, but the dose does not seem to affect the rate of onset, duration, or severity of the symptoms [9, 10]. Patient's past psychiatric history or age also appears to be unrelated to development of neurological symptoms [10]. Treatment for these side effects includes tapering of steroids and adding mood stabilizers (lithium, Valproic acid) or adding low doses of neuroleptics (haloperidol) [1, 2].

It is important to distinguish primary mania from corticosteroid-induced mania in a patient with history of mental disorder who is on steroids. As incidence of type 1 bipolar disorder is 1.8%, many people with this disorder are on steroids [10]. A number of published reports describe mood disorder as a more prominent feature of steroid psychosis than psychosis and also higher incidence of psychotic features in corticosteroid-induced mania than in primary mania [5, 11]. When someone gets the diagnosis of steroid-induced psychosis it is easy to overlook bipolar disorder. Psychiatrists have long struggled with patients who do not fit clearly into diagnostic criteria.

## 4. Conclusion

In our case a patient with history of bipolar II disorder stable on medications switched into mania when started on 60 mg/day of prednisone. Her manic symptoms resolved after discontinuation of prednisone. So this proves a stable patient on medications switched to mania because of steroids. In our paper we did not find many cases in which stable bipolar patient switched to mania on clinical doses of prednisone.
